# Clinical outcomes according to the timing of the first tracheostomy tube change

**DOI:** 10.1016/j.heliyon.2024.e28180

**Published:** 2024-03-19

**Authors:** Sangho Lee, Sang-Wook Lee, Joyoung Park, Jihoon Han

**Affiliations:** a Department of Anesthesiology and Pain Medicine, Kyung Hee University College of Medicine, Kyung Hee University Medical Center, Seoul, Republic of Korea; b Department of Anesthesiology and Pain Medicine, Asan Medical Center, University of Ulsan College of Medicine, Seoul, Republic of Korea

**Keywords:** Tracheostomy, First tracheostomy tube change, Clinical outcomes, Complications, Critical care, Mortality

## Abstract

**Purpose:**

The first tracheostomy tube replacement is a critical procedure that can cause various complications, but there are few studies on the optimal timing of tracheostomy tube replacement in adult patients. This study aimed to evaluate the appropriate timing to replace the first tracheostomy tube to improve outcomes in adult patients.

**Materials and methods:**

This study was a retrospective cohort study that included 3957 patients aged ≥18 years who underwent the first tracheostomy tube change from January 2010 to February 2021. The primary outcome was all-cause mortality after the first tracheostomy tube change.

**Results:**

The all-cause mortality was statistically significantly lower in group changing the first tracheostomy tube between 7 and 9 days than in other groups (42.1%, *P* = 0.001). After adjustments in the multivariable analyses, early first tracheostomy tube change within 6 days was independently associated with increased all-cause mortality. The hospital stay, ICU stay, and post-procedural pulmonary complications seemed to increase as the replacement time was delayed.

**Conclusions:**

The timing of the first tracheostomy tube change between 7 and 9 days after tracheostomy was associated with improved clinical outcomes, including all-cause mortality. Further prospective investigations are needed to determine whether the optimal timing of the first tracheostomy tube change can reduce mortality.

## Highlights

• 1st tracheostomy tube change can cause various complications.
• All-cause mortality decreased in changing 1st tracheostomy tube between 7 and 9 days.
• All-cause mortality increased when changing 1st tracheostomy tube within 6 days.
• Hospital stay, pulmonary complications increased as replacement time was delayed.
• Optimal timing of 1st tracheostomy tube change was associated with clinical outcome.

## Abbreviations

IRB: institutional review board
BMI: body mass index
CCI: charlson comorbidity index
ICU: intensive care unit
CT: computed tomography

## 1 Introduction

Tracheotomy is performed in the upper airway to remove secretions, resolve upper airway obstruction, require mechanical ventilation for a long period, or wean from mechanical ventilation [[1], [2], [3]]. A tracheostomy tube is placed for a considerable time period to maintain stable patency from the skin to the trachea. Compared with tracheal intubation, a tracheostomy can reduce the energy consumed for respiration, minimize damage to the larynx, improves oral hygiene, and enables oral intake [4]. However, there may be complications associated with a tracheotomy, such as emphysema, infection, tracheostomy tube obstruction, displacement, bleeding, and aspiration [5].

The tracheostomy tube is replaced for various reasons, including adjustments to the tube's size for bronchoscopy procedures, incorrect positioning due to unsuitable length or size, and the specific type of the tracheostomy tube. In addition, the first replacement is performed 7–14 days after the first tracheostomy without any particular reason, and then the replacement is performed regularly [6]. However, even when the first tracheostomy tube is changed, failure to secure the airway could lead to mortality [7], and this risk may increase if body mass index or the patient's neck circumference increases [8]. Moreover, it has been reported that if the replaced tracheostomy tube is incorrectly positioned in the anterior mediastinum, a large amount of subcutaneous emphysema, mediastinal emphysema, cardiac arrest, and in rare cases, damage to the innominate artery may occur [8]. In addition, there were studies regarding early change of the first tracheostomy tube in pediatric patients that can improve outcomes, such as fewer significant peristomal wounds, reduction intensive care unit stay, and reduction overall hospital stay [[9], [10], [11]]. However, studies about complications in adult patients remain insufficient.

Therefore, the clinician can be expected to perform more safely in the first tracheostomy tube change if the appropriate timing, which reduces complications, can be known. In this study, we hypothesized that the type and frequency of complications would be different according to the timing of the first tracheostomy tube change. Therefore, we would like to determine the safest timing of the first tracheostomy tube change based on this assumption.

## 2 Material and methods

### 2.1 Study design

This study was a single-institutional, retrospective cohort analysis involving 3957 patients aged 18 and older who underwent the first tracheostomy tube change at a tertiary medical center from January 2010 to February 2021. The study followed the guidelines outlined in the Declaration of Helsinki and was sanctioned by the institutional review board (IRB) at Asan Medical Center (Seoul, Korea, approval number 2021-0874, approval date June 11, 2021, chairperson Professor Moo-Song Lee). The IRB of Asan Medical Center waived the need for informed consent given the study's retrospective and anonymized characteristics.

### 2.2 Patients

**Fig. 1:**
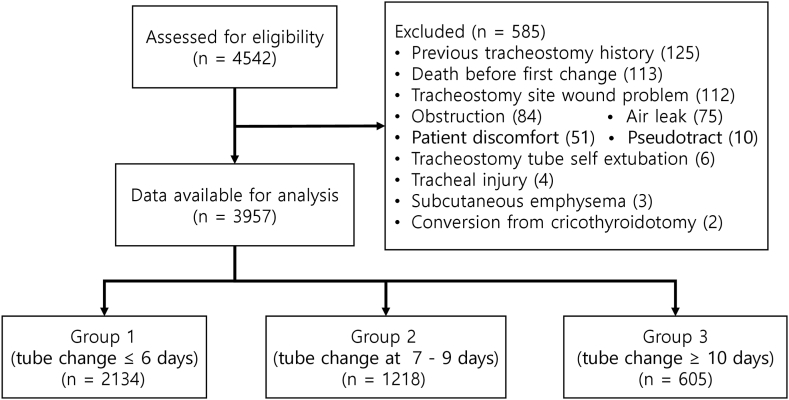
Diagram of the patient flow chart.

The inclusion criteria were patients aged ≥18 years who underwent their first tracheostomy tube change from January 2010 to February 2021. Patients were excluded if they died before the first tracheostomy tube change. The revision tracheostomy, performed again after the recovery of the first tracheostomy insertion site, was also excluded. Additionally, exclusion criteria include cases where the tracheostomy tube replacement was performed due to an inevitable situation not according to a regular schedule, such as tracheostomy site bleeding or wound problem, obstruction due to clot or mucus, patient discomfort due to persistent cough, air leak, pseudo tract formation, tracheostomy tube was pulled out spontaneously, a cricothyroidotomy was changed to a tracheostomy, in case of trachea injury occurred such as a tracheoesophageal fistula, and when subcutaneous emphysema occurred. A total of 3957 patients were divided into three groups according to the timing of the first tracheostomy tube change (ternary grouping). In group 1, 2134 patients had their tracheostomy tube changed at 6 days or less, group 2 had 1218 patients who had their tube changed between 7 and 9 days, and group 3 had 605 patients who had their tube changed at 10 days or more (Fig. 1). In addition, to clearly show the effect of an interesting time period for the first tracheostomy tube change on clinical outcomes, we used binary grouping, which was conducted by grouping the time between 7 and 9 days into one group and the remaining time period into another group (binary grouping).

### 2.3 Pre-procedural and post-procedural variables

All demographic and clinical information was obtained from the institution's electronic medical records. Pre-procedural factors included patient sex, age, body mass index (BMI), Charlson comorbidity index (CCI), time for the first tracheostomy tube change, and the cause of first tracheostomy tube change. Post-procedural variables included survivor time, death data, and cause of death.

## 3 Primary outcomes and secondary outcomes

The primary post-procedural outcome was all-cause mortality. Secondary outcomes included all-cause 30-day mortality, hospital stay, length of intensive care unit (ICU) stay, and post-procedural pulmonary complications after the first tracheostomy tube change.

Post-procedural pulmonary complications were defined as the occurrence of respiratory complications according to the keywords in the formal reading of chest X-ray and chest computed tomography (CT) conducted after the first tracheostomy tube change, such as pneumothorax, emphysema, respiratory infection, pneumonia, aspiration pneumonitis, atelectasis, tracheal stenosis, and bronchospasm.

### 3.1 Statistical analysis

Continuous variables were presented as mean values and standard deviations, while categorical variables were expressed in counts and percentages. For comparison of continuous variables between two groups, either the Student's t-test or the Mann–Whitney *U* test was utilized depending on suitability. In contrast, the chi-square test or Fisher's exact test was applied for comparing categorical variables. According to the time for first tracheostomy tube change, survival analysis was performed using the Kaplan-Meier method to plot a survival curve. Univariate and multivariate Cox proportional hazards regression analyses were conducted to identify factors impacting mortality, including the timing of the first tracheostomy tube change in relation to all-cause mortality. The hazard ratio of the Cox regression model was expressed as a 95% confidence interval. Variables that yielded *P*-values less than 0.05 in univariate Cox regression analysis, along with those deemed clinically significant, were chosen for inclusion in the multivariate Cox regression analysis. All statistical analyses were carried out using the “R” statistical software package (version 3.6.3, R Foundation for Statistical Computing, Vienna, Austria), considering a *P*-value of less than 0.05 as indicative of statistical significance.

## 4 Results

Out of 4542 consecutive patients, 585 patients were excluded; 125 patients had a history of prior tracheostomy; 113 patients died before the first tracheostomy tube change; 112 patients had wound problems such as tracheostomy site bleeding; 84 patients occurred tracheostomy tube obstruction due to clot or mucus; 75 patients occurred air leak; 51 patients complained of discomfort due to persistent coughing; 10 patients with formation of pseudo tract; 6 patients, the tracheostomy tube was pulled out spontaneously; 4 patients occurred trachea injury such as a tracheoesophageal fistula; 3 patients with formation of subcutaneous emphysema; and 2 patients converted from cricothyroidotomy (Fig. 1).

The demographic characteristics and clinical outcomes across the three groups are presented in Table 1. In demographic data, sex, age, and BMI were not significantly different between the three groups. However, CCI, which indicates the degree of comorbidity, was significantly higher in group 3 than in other groups (6.1 vs. 6.4 vs. 7.0, *P* = 0.007, Table 1).

**Table 1:** Baseline demographic data and clinical outcomes of patients who underwent first tracheostomy tube change at 6 days or less (Group 1) or change at 7–9 days (Group 2) or change at 10 days or more (Group 3). Values are presented as the mean ± standard deviation or number (percentage).

Variables	Total (n = 3957)	Group 1 (n = 2134)	Group 2 (n = 1218)	Group 3 (n = 605)	*P*-Value
1st T-can change time (days)	7.5 ± 7.6	4.9 ± 1.3	7.4 ± 0.7	17.0 ± 16.1	<0.001
Male (sex)	2663 (67.3)	1446 (67.8)	811 (66.6)	406 (67.1)	0.779
Age (years)	63.8 ± 14.2	63.9 ± 14.0	63.7 ± 14.6	63.7 ± 14.4	0.910
BMI (kg/m^2^)	22.6 ± 4.2	22.5 ± 4.1	22.7 ± 4.2	22.7 ± 4.3	0.395
CCI	6.3 ± 6.6	6.1 ± 6.2	6.4 ± 6.8	7.0 ± 7.2	0.007
Post-procedural pulmonary Cx	3214 (81.2)	1696 (79.5)	1013 (83.2)	505 (83.5)	0.010
Hospital stay (days)	90.0 ± 216.8	78.6 ± 179.0	94.6 ± 218.4	120.9 ± 311.8	<0.001
ICU stay (days)	36.0 ± 47.9	32.0 ± 39.9	37.6 ± 49.7	47.0 ± 65.2	<0.001
All-cause mortality	1829 (46.2)	1043 (48.9)	513 (42.1)	273 (45.1)	0.001
30-day mortality	655 (16.6)	368 (17.2)	188 (15.4)	99 (16.4)	0.395
Cause of death					0.003
Respiratory failure	962 (24.3)	567 (26.6)	249 (20.4)	146 (24.1)	
Shock	347 (8.8)	185 (8.7)	120 (9.9)	42 (6.9)	
Cancer	331 (8.4)	181 (8.5)	92 (7.6)	58 (9.6)	
CVA	98 (2.5)	60 (2.8)	26 (2.1)	12 (2.0)	
Metabolic disorder	8 (0.2)	5 (0.2)	0	3 (0.5)	
Liver failure	78 (2.0)	42 (2.0)	24 (2.0)	12 (2.0)	
Unknown	5 (0.1)	3 (0.1)	2 (0.2)	0	

T-can: tracheostomy tube. BMI: body mass index. CCI: Charlson comorbidity index. Cx.: complication. ICU: intensive care unit. CVA: cerebrovascular accident.

The all-cause mortality was the lowest in group 2 and the highest in group 1 at a statistically significant level (48.9% vs. 42.1% vs. 45.1%, *P* = 0.001, Table 1). Among the causes of death, respiratory failure was the lowest in group 2, while the highest in group 1 (26.6% vs. 20.4% vs. 24.1%, *P* = 0.003, Table 1). The 30-day mortality after the first tracheostomy tube change was the lowest in group 2 and the highest in group 1, but it was not statistically significant. The post-procedural pulmonary complications confirmed by the formal readings on radiographic examinations increased as the time to change the tracheostomy tube for the first time was delayed (79.5% vs. 83.2% vs. 83.5%, *P* = 0.010, Table 1). The length of hospital stay (78.6 vs. 94.6 vs. 120.9, *P* < 0.001, Table 1) and ICU stay (32.0 vs. 37.6 vs. 47.0, *P* < 0.001, Table 1) also tended to increase as the tube changed time was delayed.

Univariable Cox regression analysis of the factors that affected the all-cause mortality after the first tracheostomy tube change showed that the time for the first tracheostomy tube change (≤6 days and ≥10 days, binary grouping), sex, age, BMI, and CCI were significantly associated with the all-cause mortality (Table 2). Multivariable Cox regression analysis included the significant factors and variables derived from the univariable Cox analysis and found that BMI and CCI, including the first tracheostomy tube change time, were significantly related to the all-cause mortality (Table 2).

**Table 2:** Univariable and multivariable cox regression analysis of factors associated with all-cause mortality after first tracheostomy tube change.

		Univariable		Multivariable	
		Unadjusted HR (95% CI)	*P*-Value	Adjusted HR (95% CI)	*P*-Value
Sex (male)		1.21 (1.09, 1.34)	<0.001		
Age		1.01 (1.01, 1.02)	<0.001		
BMI		0.98 (0.97, 0.99)	<0.001	0.98 (0.97, 0.99)	<0.001
CCI		1.06 (1.06, 1.07)	<0.001	1.06 (1.06, 1.07)	<0.001
1st T-can change time					
Ternary grouping	≤6 d	1.11 (1.00, 1.23)	0.060	1.16 (1.04, 1.29)	0.007
	7–9 d	1 (reference)		1 (reference)	
	≥10 d	1.12 (0.97, 1.30)	0.120	1.08 (0.94, 1.26)	0.286
Binary grouping	7–9 d	1 (reference)		1 (reference)	
	≤6 d & ≥ 10 d	1.11 (1.00, 1.23)	0.044	1.14 (1.03, 1.27)	0.011

HR: hazard ratio. CI: confidence interval. T-can: tracheostomy tube. BMI: body mass index. CCI: Charlson comorbidity index. Cx.: complications. ICU: intensive care unit.

After adjusting for sex, age, BMI, and CCI in the multivariable analyses, early first tracheostomy tube change at 6 days or less in the ternary grouping for the tube change time was determined to be independently associated with increased all-cause mortality, while there were no statistically significant associations between late first tracheostomy tube change at 10 days or more and all-cause mortality (Table 2). However, in the binary grouping for tube change time, except for the period between 7 and 9 days, the mortality rate was statistically significantly increased before and after adjusted by sex, age, BMI, and CCI (Table 2).

**Fig. 2:**
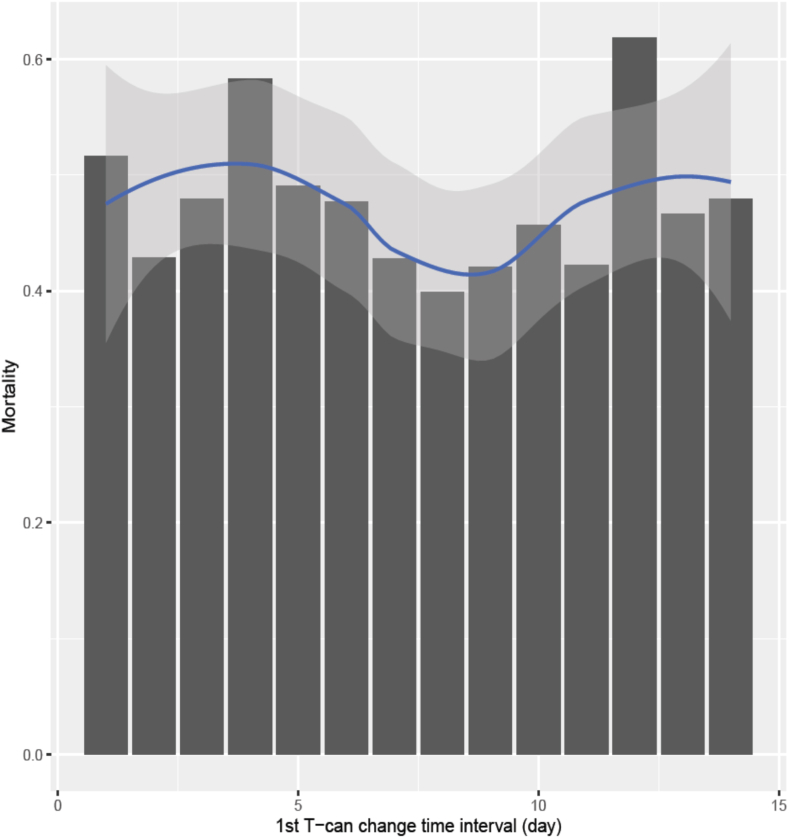
Mortality rate according to timing of the first tracheostomy tube change.

The timing of the first tracheostomy tube change appeared to have a U-shaped hazard function for all-cause mortality. In other words, when the first tube change was performed early or too late, the all-cause mortality was increased (Fig. 2).

**Fig. 3:**
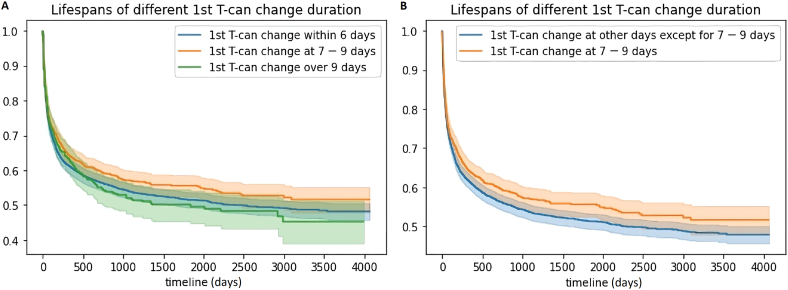
(A) Kaplan-Meier analysis of overall survival in three groups of patients classified by first tracheostomy tube change timing within 6 days or at 7–9 days or over 9 days. (B) Kaplan–Meier analysis of overall survival in two groups of patients classified by first tracheostomy tube change timing at 7–9 days or other days.

In the Kaplan–Meier analysis, the patients were divided into three groups according to the timing of the first tracheostomy tube change, the survival rate was higher in the group of the first tube change at 7–9 days than in other groups, but there was no statistically significant difference between the groups (log-rank *P* = 0.128, Fig. 3A). Alternatively, when the patients were divided into two groups with first tube change at 7–9 days or others except for 7–9 days, the group with tube change at 7–9 days had a higher survival rate, and there was a statistically significant difference between the two groups (log-rank *P* = 0.044, Fig. 3B).

## 5 Discussion

In this study, 3957 patients who underwent tracheostomy were analyzed for the prognosis according to the timing of the first tracheostomy tube change. The present study demonstrated that mortality varies according to the timing of the first tracheostomy tube change. The tube replacement at 7–9 days was associated with reduced all-cause mortality compared to other change schedules. In addition, the mortality due to respiratory failure also decreased in the group of tube changes at 7–9 days. However, the 30-day mortality rates did not show statistically significant differences among the three groups. In addition, the post-procedural pulmonary complications, hospital stay, and ICU stay seemed to increase when tube change was delayed.

Most previous studies have focused on the first tracheostomy tube change in pediatric patients [12]. In most studies, the first tracheostomy tube is changed within approximately 2–4 days, and there are no respiratory complications, wound problems, and healing; thus, an early replacement was recommended [[9], [10], [11],^13^]. However, there may be differences in wound healing between adults and pediatric patients. In general, it is known that wound healing is faster in children due to the reduction of granulation tissue and the inflammatory phase [14,15]. Therefore, when the early tube change is performed in adult patients as in children, delayed wound healing can cause problems; thus, it is recommended to avoid the early first tube change.

In previous studies related to adult patients who underwent tracheostomy, there were much research on when to perform tracheostomy itself, and it was mainly recommended to perform tracheostomy at an early stage of each medical conditions [16,17]. Brenner et al. [18] reported that multidisciplinary teamwork, standardization, education, and patient partnership were important for improving the safety of patients with a tracheostomy. McGrath et al. [19] reported that the mortality, length of stay, and quality of life were improved in a large-scale prospective study applying the aforementioned items. In the current large-scale retrospective study, we could not evaluate multidisciplinary teamwork, standardization, education, and patient partnership related to individual tracheostomy tube replacement. However, in current study, we focused on when to perform the first tracheostomy tube change, and it is meant to suggest an appropriate change timing so that a safe replacement of the tracheostomy tube can be performed. Clinically, the first tracheostomy tube change is an important issue that must not be overlooked after tracheostomy is performed. Although this was a retrospective study and we could not suggest causality between the first tracheostomy tube change and clinical outcomes, our findings suggested an association between them.

An increase in the patient's neck circumference and BMI is a risk factor for performing tracheostomy and changing the tracheostomy tube [8,20,21]. In this retrospective analysis, only the patient's BMI was considered, which resulted in no significant differences across the three groups. However, Cox regression analysis showed that the lower the BMI, the higher the all-cause mortality. This could be interpreted as the deterioration of the general condition when BMI decreased had a greater effect on the all-cause mortality than the increase in BMI, resulting in an increased risk of tracheostomy tube change [22].

The post-procedural pulmonary complications increased as the first tube change was delayed. In the case of delayed tracheostomy tube change, reports have demonstrated that complications increase due to the contamination of the tube strap and delayed detection of wound problems [10,11]. However, in this study, post-procedural pulmonary complications were determined only with the presence or absence of abnormal findings on the formal reading such as pneumothorax, emphysema, respiratory infection, pneumonia, aspiration pneumonitis, atelectasis, tracheal stenosis, and bronchospasm. This study did not consider the severity of the complications; thus, it could be regarded as having no clinical significance. Hospital stay and ICU stay tended to increase as the replacement time was delayed. In several previous studies, it is known that the length of stay increases as the CCI increases [23,24]. In this study, as the CCI increased, the change of the first tracheostomy tube tended to be delayed and thought to be due to other comorbid diseases. In addition, the length of hospital and ICU stay has increased due to the increase in CCI. The 30-day mortality showed a similar trend to the all-cause mortality, but it did not show a statistically significant difference. Therefore, a meaningful difference can be seen when the sample size increases.

Early replacement within 6 days was analyzed to have a worse effect on the all-cause mortality when the tube change timing on all-cause mortality was adjusted by sex, age, BMI, and CCI. On the other hand, a delayed replacement for more than 10 days had no significant effect on all-cause mortality. Generally, it takes 5–10 days for the stoma to mature after performing a tracheostomy [25,26]. Therefore, it showed the risk of early tracheostomy tube replacement at a time when the stoma is not mature, and caution is highly required if the tracheostomy tube is replaced early due to unavoidable reasons.

Our study has several strengths and clinical significance in several areas. First, our study is one of the rare studies that provide important lessons on the timing of the first tracheostomy tube change. Most of the previous studies were in pediatric patients or small studies with very small number of patients [[9], [10], [11]]. Therefore, we can say that this study is valuable in dealing with relatively large-scaled data and the study of adult patients. Moreover, this study was a large-scale study targeting non-western patients in a single tertiary medical center.

There are several limitations to this study. Given its retrospective nature, this study may not offer the same level of clinical significance as a prospective randomized controlled trial, and causality could not be explained between the first tracheostomy tube change and clinical outcomes, and each group's sample size was not uniform. Since a wide range of factors over various time periods affect all-cause mortality, it is difficult to control the influencing factors due to the limitations of a retrospective study. However, to overcome these limitations, we applied strict inclusion criteria to exclude peri-procedural factors that may affect mortality from the study. In addition, CCI that reflects the comorbidity of patients, sex, age, and BMI were adjusted for the effect of tube change time on all-cause mortality using multivariable Cox regression analysis. However, confounding variables such as difficult airway, hypertension, and hyperlipidemia, which were not included in the CCI, were not considered. In addition, data for approximately 11 years, including the COVID-19 pandemic, changes in clinical protocols, equipment development, and improvement in operator skill may affect the interpretation of the results. Even during the pandemic, there were no significant changes to the procedures of tracheostomy tube change and no increase in mortality. Last, because this was a large-scale retrospective study, discrimination between techniques for tracheostomy insertion, such as the presence or absence of a flap, could not be confirmed for individual patients. Despite these limitations, this retrospective analysis based on large-scale data may be meaningful because it is difficult to perform randomized control trials due to ethical problems with the nature of the research participants.

## 6 Conclusion

In conclusion, this study showed that the timing of the first tracheostomy tube change between 7 and 9 days after tracheostomy was associated with improved clinical outcomes, including all-cause mortality. These findings suggest that an appropriate first tracheostomy tube change timing can improve clinical outcomes. This study is a very clinically significant study in that it suggested an optimal timing to change the first tracheostomy tube in critical care adult patients with tracheostomy. However, prospective randomized trials in the future are needed to secure more reliable evidence to find a convincing optimal timing for the first tracheostomy tube change.

## Funding

This study was supported by a grant (2023IP0132-1, 2023IP0132-2) from the Asan Institute for Life Sciences, Asan Medical Center, Seoul, Korea. This study was also supported by a grant of the Korea Health Technology R&D Project through the Korea Health Industry Development Institute (KHIDI), funded by the Ministry of Health & Welfare, Republic of Korea (grant numbers: HR20C0026, HI22C1723).

## Ethics statement

The study followed the guidelines outlined in the Declaration of Helsinki and was sanctioned by the institutional review board (IRB) at Asan Medical Center (Seoul, Korea, approval number 2021-0874, approval date June 11, 2021, chairperson Professor Moo-Song Lee). The IRB of Asan Medical Center waived the need for informed consent given the study's retrospective and anonymized characteristics.

## Data availability statement

The datasets employed and analyzed during this study are accessible from the corresponding author upon reasonable request. They are not publicly available due to considerations of privacy and ethics.

## CRediT authorship contribution statement

**Sangho Lee:** Writing – review & editing, Writing – original draft, Methodology, Data curation. **Sang-Wook Lee:** Writing – review & editing, Writing – original draft, Visualization, Validation, Supervision, Methodology, Funding acquisition, Formal analysis, Data curation, Conceptualization. **Joyoung Park:** Data curation. **Jihoon Han:** Data curation.

## Declaration of competing interest

The authors declare that they have no known competing financial interests or personal relationships that could have appeared to influence the work reported in this paper.
